# Mossy cell hypertrophy and synaptic changes in the hilus following mild diffuse traumatic brain injury in pigs

**DOI:** 10.1186/s12974-020-1720-0

**Published:** 2020-01-31

**Authors:** Michael R. Grovola, Nicholas Paleologos, Kathryn L. Wofford, James P. Harris, Kevin D. Browne, Victoria Johnson, John E. Duda, John A. Wolf, D. Kacy Cullen

**Affiliations:** 1 Center for Neurotrauma, Neurodegeneration & Restoration, Corporal Michael J. Crescenz VA Medical Center, Philadelphia, PA USA; 2 Center for Brain Injury & Repair, Department of Neurosurgery, University of Pennsylvania, 105E Hayden Hall/3320 Smith Walk, Philadelphia, PA 19104 USA; 3 School of Biomedical Engineering, Science and Health Systems, Drexel University, Philadelphia, PA USA; 4 Parkinson’s Disease Research, Education and Clinical Center, Corporal Michael J. Crescenz VA Medical Center, Philadelphia, PA USA; 5 Department of Neurology, Perelman School of Medicine, University of Pennsylvania, Philadelphia, PA USA; 6 Department of Bioengineering, School of Engineering and Applied Science, University of Pennsylvania, 105E Hayden Hall/3320 Smith Walk, Philadelphia, PA 19104 USA

**Keywords:** Mild traumatic brain injury, Concussion, Microglia, Hippocampus, Mossy cell

## Abstract

**Background:**

Each year in the USA, over 2.4 million people experience mild traumatic brain injury (TBI), which can induce long-term neurological deficits. The dentate gyrus of the hippocampus is notably susceptible to damage following TBI, as hilar mossy cell changes in particular may contribute to post-TBI dysfunction. Moreover, microglial activation after TBI may play a role in hippocampal circuit and/or synaptic remodeling; however, the potential effects of chronic microglial changes are currently unknown. The objective of the current study was to assess neuropathological and neuroinflammatory changes in subregions of the dentate gyrus at acute to chronic time points following mild TBI using an established model of closed-head rotational acceleration induced TBI in pigs.

**Methods:**

This study utilized archival tissue of pigs which were subjected to sham conditions or rapid head rotation in the coronal plane to generate mild TBI. A quantitative assessment of neuropathological changes in the hippocampus was performed via immunohistochemical labeling of whole coronal tissue sections at 3 days post-injury (DPI), 7 DPI, 30 DPI, and 1 year post-injury (YPI), with a focus on mossy cell atrophy and synaptic reorganization, in context with microglial alterations (e.g., density, proximity to mossy cells) in the dentate gyrus.

**Results:**

There were no changes in mossy cell density between sham and injured animals, indicating no frank loss of mossy cells at the mild injury level evaluated. However, we found significant mossy cell hypertrophy at 7 DPI and 30 DPI in anterior (> 16% increase in mean cell area at each time; *p* = <  0.001 each) and 30 DPI in posterior (8.3% increase; *p* = <  0.0001) hippocampus. We also found dramatic increases in synapsin staining around mossy cells at 7 DPI in both anterior (74.7% increase in synapsin labeling; *p* = <  0.0001) and posterior (82.7% increase; *p* = <  0.0001) hippocampus. Interestingly, these morphological and synaptic alterations correlated with a significant change in microglia in proximity to mossy cells at 7 DPI in anterior and at 30 DPI in the posterior hippocampus. For broader context, while we found that there were significant increases in microglia density in the granule cell layer at 30 DPI (anterior and posterior) and 1 YPI (posterior only) and in the molecular layer at 1 YPI (anterior only), we found no significant changes in overall microglial density in the hilus at any of the time points evaluated post-injury.

**Conclusions:**

The alterations of mossy cell size and synaptic inputs paired with changes in microglia density around the cells demonstrate the susceptibility of hilar mossy cells after even mild TBI. This subtle hilar mossy cell pathology may play a role in aberrant hippocampal function post-TBI, although additional studies are needed to characterize potential physiological and cognitive alterations.

## Background

Every year, over 2.4 million people experience mild traumatic brain injury (TBI) [1]. Recent studies suggest that even mild TBI may be associated with long-term neurological deficits such as post-concussive syndrome and epilepsy [2, 3]. One previous study using the current injury model has revealed synaptic dysfunction and regional hyperexcitability in the hippocampus, a structure essential for memory formation, after a single mild TBI [4]. Indeed, other studies have shown that mild TBI may induce neuropathological changes in the hippocampus, with the hilar region showing particular vulnerability to cell loss or aberrant axonal sprouting in preclinical models of TBI [5–8]. Mossy cells, the principal glutamatergic neuron of the hilus, are of particular interest due to their hallmark loss in temporal lobe epilepsy and potential role in post-TBI dysfunction [5, 9, 10].

In addition, evidence suggests critical interplay between neuronal dysfunction, synaptic remodeling, and inflammatory processes. For example, recent studies on synaptic component changes and the development of neuronal hyperexcitability suggest that inflammation may be a contributing factor [11]. Genes associated with proinflammatory pathways are upregulated in patients with temporal lobe epilepsy and hippocampal sclerosis [12]. Additionally, human patients with temporal lobe epilepsy and hippocampal sclerosis, as well as rodents in experimental models of epilepsy, have displayed histopathological activation of macrophages, the primary peripheral innate immune cells that can access the brain following an insult, and microglia, the resident immune cells of the central nervous system [13]. In particular, rodent epilepsy studies have demonstrated that microglial processes are recruited by hyperactive neurons [14, 15]. Importantly, synaptic inputs can be modified by microglia in both healthy and disease states: microglia have been shown to engulf and eliminate synapses during normal neuronal development [16–19], as well as during ongoing adult neurogenesis [20] and the progression of Alzheimer’s disease [21]. While the precise role of microglia following TBI is still unclear, activated microglia have been shown in areas of neuronal damage in both human and animal models [22–25].

Overall, previous research has suggested that the hilus is vulnerable after TBI in other models, and that changes in hilar mossy cells may contribute to aberrant hippocampal function post-TBI. Therefore, using a rotation-acceleration closed head injury model in pigs, the current study seeks to histologically examine synaptic changes and microglial activity around hilar mossy cells. We hypothesized that microglia will be more active in the hippocampal hilus and that there will be mossy cell loss following mild TBI.

## Methods

All procedures were approved by the Institutional Animal Care and Use Committees at the University of Pennsylvania and the Michael J. Crescenz Veterans Affairs Medical Center and adhered to the guidelines set forth in the NIH Public Health Service Policy on Humane Care and Use of Laboratory Animals (2015).

Specimens used for the current study were from a tissue archive derived from castrated male pigs subjected to a single mild TBI. These 5–6-month-old, sexually mature (considered to be young adult), Yucatan miniature pigs at a mean weight of 34 ± 4 kg (*n* = 28, mean ± SEM) were fasted for 16 h then anesthesia was induced with 20 mg/kg of ketamine and 0.5 mg/kg of midazolam. After induction, 0.1 mg/kg of glycopyrrolate was subcutaneously administered and 50 mg/kg of acetaminophen was administered per rectum. All animals were intubated with an endotracheal tube and anesthesia was maintained with 2% isoflurane per 2 l O_2_. Heart rate, respiratory rate, arterial oxygen saturation, and temperature were continuously monitored throughout the experiment. A forced-air temperature management system was used to maintain normothermia throughout the procedure.

**Fig. 1:**
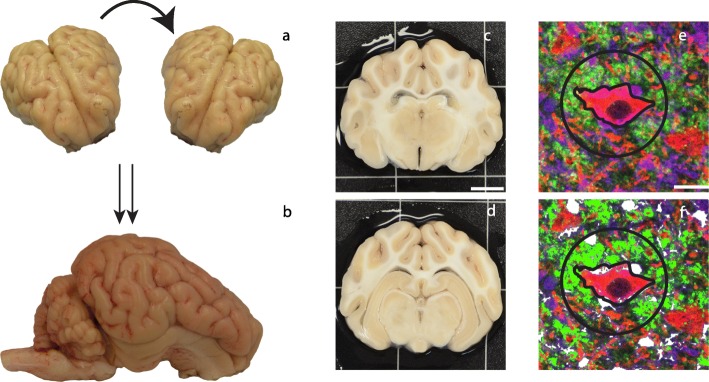
Injury model and analysis methodology. **a** A rostral view of an extracted pig brain. The arrow demonstrates the coronal plane rotation. **b** A sagittal view of an extracted pig brain. The right arrow shows the location of the anterior hippocampal tissue (**c**; scale bar = 1 cm), while the left arrow shows the location of the posterior hippocampal tissue (**d**). **e** All tissue was imaged on a confocal microscope for Hoechst (blue), synapsin (green), MAP2 (red), and Iba-1 (purple). After imaging, each mossy cell was outlined to obtain the area of each cell and a 30-μm radius circle was created around the epicenter of each mossy cell (scale bar = 20 μm). **f** Highlights the extent of positive pixels detected by the Nikon Elements software, which were analyzed for the current study. Synapsin-positive pixels are shown in neon green and Iba-1-positive pixels are shown in white

In order to attain closed-head diffuse mild TBI in animals, we used a previously described model of head rotational-acceleration in pigs [26, 27]. Briefly, each animal’s head was secured to a bite plate, which itself was attached to a pneumatic actuator and a custom assembly that converts linear motion into angular momentum. The pneumatic actuator rotated each animal’s head in the coronal plane at a target angular velocity of 260 rad/s (Fig. 1a). Any presence of apnea was recorded and animals were hemodynamically stabilized if necessary. Sham animals (*n* = 13) underwent identical protocols, including being secured to the bite plate; however, the pneumatic actuator was not initiated. All animals were transported back to their housing facility, monitored acutely for 3 h, and given access to food and water. Afterwards, animals were monitored daily for 3 days by veterinary staff.

At 3 days post-injury (DPI) (*n* = 4), 7 DPI (*n* = 5), 30 DPI (*n* = 3), or 1 year post-injury (YPI) (*n* = 3), animals were induced and intubated as described above. Sham animals survived for 0 days (*n* = 1), 7 days (*n* = 4), 14 days (*n* = 2), 30 days (*n* = 1), or 1 year (*n* = 5). While under anesthesia, animals were transcardially perfused with 0.9% heparinized saline followed by 10% neutral buffered formalin (NBF). Animals were then decapitated and tissue stored overnight in 10% NBF at 4 °C. The following day, the brain was extracted and post-fixed in 10% NBF at 4 °C for 1 week. To block the tissue, an initial coronal slice was made immediately rostral to the optic chiasm. The brain was then blocked into 5-mm-thick coronal sections from that point by the same investigator. This allowed for consistent blocking and section coordinates across animals. All blocks of tissue were paraffin embedded and 8-μm-thick sections were obtained using a rotary microtome. Two sections from each specimen—one containing anterior aspects of hippocampal tissue (approximately 10 mm posterior to the optic chiasm) and one containing posterior aspects of hippocampal tissue (approximately 15 mm posterior to the optic chiasm)—were used for the ensuing histological analysis. Though their functionality in the hippocampus cannot be distinguished at this time, the anterior and posterior aspects of hippocampal tissue have different gross anatomies and the forces induced by closed-head TBI may act differently on these regions. Anterior and posterior sections were used to provide more histological insight and be more representative of the hippocampus as a whole (Fig. 1c, d).

For fluorescence immunohistochemical labeling, slides were dewaxed in xylene, and rehydrated in ethanol and deionized water. Antigen retrieval was completed in Tris EDTA buffer pH 8.0 using a microwave pressure cooker then blocked with normal horse serum. Slides were incubated overnight at 4 °C using anti-mouse synapsin I (Synaptic Systems, 106-001, 1:5000), anti-chicken Microtubule Associated Protein 2 (MAP2) (Abcam, ab5392, 1:1000), and anti-rabbit Iba-1 (Wako, 019-19741, 1:1000) primary antibodies. The following day, sections were rinsed in PBS and incubated in donkey anti-mouse 488 (Invitrogen, A21202, 1:500), donkey anti-chicken 594 (Jackson Laboratories, 703-585-155, 1:500), and donkey anti-rabbit 647 (Invitrogen, A31573, 1:500) secondary antibodies for 60 min. Sections were rinsed again, then incubated with Hoechst (Life Technologies, H3570, 1:10000) to visualize DNA, and finally, coverslipped using Fluoromount-G (Southern Biotech, 0100-01). All sections were stained in the same histological sample run. All stained sections were imaged and analyzed at × 20 optical zoom and × 1.5 digital zoom using a Nikon A1R Confocal microscope. *Z*-plane acquisition was determined by detectable fluorescent labeling; the brightest synapsin fluorescent signal in the *z*-plane was the center point for *z*-stacked images. Each acquired *z*-stack was seven steps at 1.1 μm spacing and was held consistent throughout all tissues. Each *z*-stack was then flattened to a 2D maximum projection. Representative images are shown in Fig. 3.

For 3,3′-diaminobenzidine (DAB) immunohistochemical labeling, we used a protocol outlined in Johnson et al. [28]. Briefly, slides were also dewaxed in xylene, rehydrated in ethanol, and deionized in water. Antigen retrieval was completed in Tris EDTA buffer pH 8.0 using a microwave pressure cooker then blocked with normal horse serum. Slides were incubated overnight at 4 °C using an anti-rabbit Iba-1 (Wako, 019-19741, 1:4000) primary antibody. The following day, slides were rinsed in PBS and incubated in a horse anti-mouse/rabbit biotinylated IgG secondary antibody (VECTASTAIN Elite ABC Kit, Vector Labs, PK-6200). Sections were rinsed again, then incubated with an avidin/biotinylated enzyme complex (VECTASTAIN Elite ABC Kit), rinsed again, and incubated with the DAB enzyme substrate (Vector Labs, SK-4100) for 7 min. Sections were counterstained with hematoxylin, dehydrated in ethanol, cleared in xylene, and finally coverslipped using cytoseal. All sections were stained in the same histological sample run. All sections were imaged and analyzed at × 20 optical zoom using an Aperio CS2 digital slide scanner (Leica Biosystems Inc., Buffalo Grove, IL).

For quantitative analysis, the left hemisphere hippocampal hilus of each DAB-stained specimen was used. The left hemisphere was chosen as it has generally exhibited increased axonal pathology as this is the leading hemisphere in our head rotation injury model in the coronal plane [26, 29]. All slide identifications were blinded then subregions of the hippocampus were identified: hilus, granule cell layer (GCL), and molecular layer (ML). The examined molecular layer was defined as the area between the granule cell layer and the visible perforant path fibers. The granule cell layer included all visible granule cells. The examined hilus included all regions containing visible mossy cells immediately deep to the granule cell layer. We then counted all Iba-1-positive cells in each region using the ImageJ software. Briefly, images were converted to grayscale and deconvoluted, and the “Analyze Particles” plug-in was used to count cells. Particles less than 20 μm^2^ were excluded as these tended to be DAB-stained microglial processes in the field of view, detached from a microglial cell body. Blinded, semiquantitative, morphological assessment was also conducted on these Iba-1-positive cells in each hippocampal subregion. Microglia were considered active if they had shorter, thicker processes, while non-reactive microglia had longer, fine processes. Similar to Lafrenaye et al., the degree of microglial activation was assessed using a graded scale from 0 to 3 (0 = no microglial activation observed, 1 = ramified microglia with shorter, thicker processes in less than 10% of the region, 2 = activated microglia in 10–25% of the region, 3 = activated microglia in > 25% of the region) [25].

For fluorescent specimen analysis, the left hemisphere hippocampal hilus of each specimen was again subject to quantitative analysis. All annotations and analyses were conducted blinded. Mossy cells were identified by their location in the subgranular zone of the hilus and distinguished by their larger size and larger synaptic clouds compared to interneurons. Scharfman and Myers have proposed a list of additional criteria to identify mossy cells, such as tracing mossy cell axon innervation to the inner molecular layer as well as a series of physiological characteristics; however, these criteria could not be assessed in the archival tissue used in the current study [30]. We identified mossy cells in time-matched sham (*n* = 72), 7 DPI (*n* = 117), and 30 DPI (*n* = 89) anterior hippocampal tissue, as well as in time-matched sham (*n* = 346), 7 DPI (*n* = 349), and 30 DPI (*n* = 304) posterior hippocampal tissue. Posterior hippocampal tissue has a larger area than anterior hippocampal tissue, so a greater number of mossy cells were anticipated in posterior hippocampus. All mossy cells in the section of the tissue were included in the analysis if they had a visible soma and nucleus. Using the Nikon Elements software, a region of interest was drawn around each mossy cell with a visible soma (Fig. 1e). This allowed us to obtain the area of each mossy cell. Additionally, a 30-μm radius circle was drawn around the epicenter of each cell so that we could assess mossy cell synaptic clouds and microglia in close proximity to mossy cells. The use of a 30-μm ROI was observationally determined from a pilot study, as this ROI seemed to include all synapsin clouds and microglia changes around mossy cells. While our previous study utilized a 60-μm ROI, that study examined a different neuroanatomical area (cortex) and a different level of injury (moderate TBI), so microglial activity was anticipated to be different. The synapsin staining within the 30-μm circle was thresholded and quantified for sum intensity using the Nikon Elements software, resulting in a single synapsin sum intensity value for each identified mossy cell. Iba-1-positive microglia were counted within the 30-μm radius circle and included in the analysis if they also had visible Hoechst-positive nuclei (Fig. 1e,f).

Statistical analysis was performed using the GraphPad Prism statistical software (GraphPad Software Inc. La Jolla, CA). All immunohistochemical quantification was assessed via one-way analysis of variance (ANOVA) and Tukey’s multiple comparisons test. One-way ANOVA results are reported as *F* = (degrees of freedom numerator/degrees of freedom denominator) *F* value, *p* value). For the synapsin protein expression and mossy cell soma size analysis, the total number of cells within the defined hilar region was assessed. This approach allows for a within-subject analysis to assess the variance of soma size and synapsin expression within each individual specimen and also serves as a type of repeated measure. Nonparametric two-sample Kolmogorov-Smirnov (K-S) tests were used to compare the cumulative distribution of two datasets. Mean, standard error of the mean, and 95% confidence intervals were reported. Additionally, Cohen’s *d* serves as a standardized metric of the magnitude of the reported effects. Differences were considered significant if *p* < 0.05. As this was an archival study, power calculations were not used to determine the number of specimens for each experimental group; the current study used all available specimens exposed to a single mild TBI.

## Results

### Hilar mossy cell density did not change after mild TBI

**Fig. 2:**
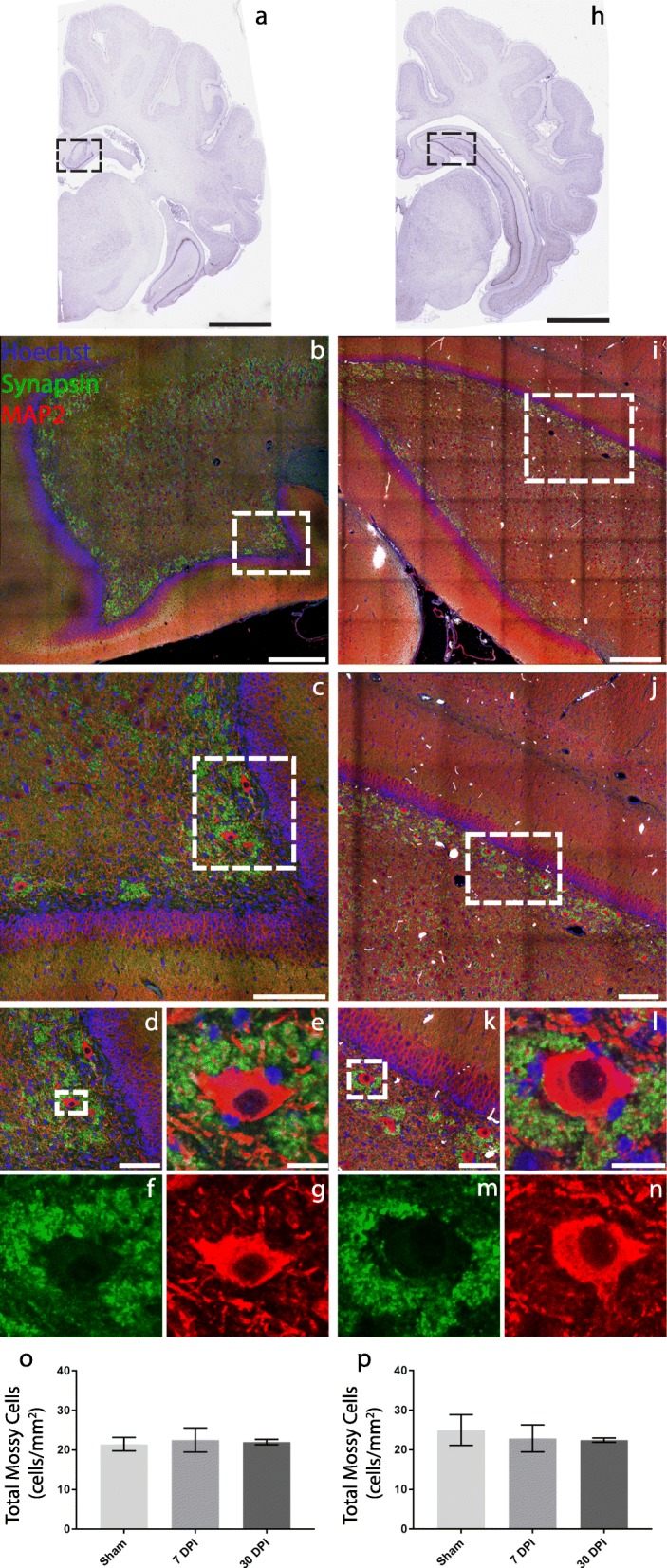
Immunohistochemical labeling reveals mossy cell morphology, but no cell loss following mild TBI. Coronal sections of pig brain containing the anterior (**a**) and posterior (**h**) levels of hippocampus used for the current analysis (scale = 1 mm). **b** The call-out box in **a** is labeled for Hoechst—a fluorescent DNA marker, synapsin—a presynaptic vesicle marker, and MAP2—a microtubule associated protein (scale = 500 μm). **c** A zoom-in of the call-out box in **b** (scale = 200 μm). **d** A zoom-in of the call-out box in **c** (scale = 100 μm). **e**–**g** A single cell view of an anterior hilar mossy cell from the call-out box in **d**. The overlay of all fluorescent labels is depicted (**e**), while the individual labels of synapsin (**f**) and MAP2 (**g**) are also shown (scale = 20 μm). **i** The call-out box in **h** is labeled for Hoechst, synapsin, and MAP2 (scale = 500 μm). **j** A zoom-in of the call-out box in **i** (scale = 250 μm). **k** A zoom-in of the call-out box in **j** (scale = 100 μm). **l**–**n** A single cell view of a posterior hilar mossy cell from the call-out box in **k**. The overlay of all fluorescent labels is depicted (**l**), while the individual labels of synapsin (**m**) and MAP2 (**n**) are also shown (scale = 20 μm). Bar graphs of the total number of mossy cells in anterior (**o**) and posterior (**p**) sections show that there is no frank loss of mossy cells following mild TBI

We performed detailed quantitative cell-level studies in pig tissue sections at 7 DPI and 30 DPI versus sham to provide an in-depth analysis of microglial and synaptic changes around hilar mossy cells following mild TBI. Although other studies have found protein-specific markers for mossy cells in rodents and humans, there is no reported marker for mossy cells in pig tissue [31, 32]. Consequently, mossy cells were distinguished from hilar interneurons by their large size, somatic and dendritic morphology, and large synaptic clouds (Fig. 2).

Previous literature suggests that TBI may initiate mossy cell death; therefore, we sought to quantify potential mossy cell loss after mild TBI in our model. In anterior hippocampal hilus (*F* = (2/9) 0.05112, *p* = 0.9504), mossy cell loss was not detected at 7 DPI (mean = 22.55 cells/mm^2^, SEM ± 3.048) (*p* = 0.9456, *d* = 0.20) or 30 DPI (mean = 22.03 cells/mm^2^, SEM ± 1.153) (*p* = 0.9885, *d* = 0.22) compared with sham (mean = 21.48 cells/mm^2^, SEM ± 1.702). There was also no evidence of mossy cell loss between 7 DPI and 30 DPI (*p* = 0.9890, *d* = 0.11) (Fig. 2o). Moreover, in the posterior hippocampal hilus, there was also no detectable mossy cell loss (*F* = (2/9) 0.1524, *p* = 0.8608). There was no significant difference in the number of mossy cells in sham (mean = 24.99 cells/mm^2^, SEM ± 3.879) compared to 7 DPI (mean = 22.90 cells/mm^2^, SEM ± 3.399) (*p* = 0.8911, *d* = 0.27) or 30 DPI (mean = 22.45 cells/mm^2^, SEM ± 0.5451) (*p* = 0.8774, *d* = 0.46). Additionally, there was no significant difference between 7 DPI and 30 DPI (*p* = 0.9955, *d* = 0.08) (Fig. 2p).

### Mossy cell hypertrophy at 7 and 30 days after mild TBI

**Fig. 3:**
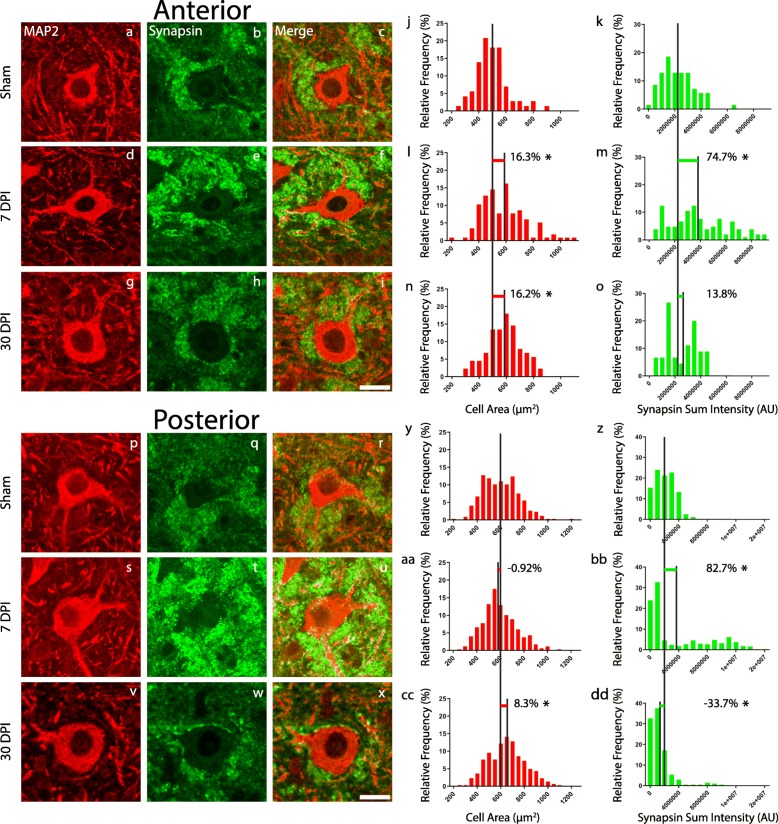
Synaptic vesicle clouds increase at 7 DPI and mossy cell somata hypertrophy following mild TBI. In anterior hilus, mossy cell soma area increased at 7 DPI (**d**) and 30 DPI (**g**) compared to sham (**a**). Histograms of mossy cell area (**j**, **l**, **n**) illustrate a rightward shift of the data distribution at 7 DPI and 30 DPI compared to sham. Sum intensity of synapsin in anterior hilus increased at 7 DPI (**e**) compared to sham (**b**). Synapsin labeling then decreased at 30 DPI (**h**) compared to 7 DPI. Histograms of synapsin sum intensity (**k**, **m**, **o**) show the frequency of higher synapsin sum intensities at 7 DPI compared to sham and 30 DPI. (**c**, **f**, **i**) display the merge of synapsin and MAP2 channels (scale bar = 25 μm). In posterior hilus, mossy cell soma area increased at 30 DPI (**v**) compared to sham (**p**) and 7 DPI (**s**). Histograms of mossy cell area (**y**, **aa**, **cc**) display a rightward shift of the data distribution at 30 DPI compared to sham and 7 DPI. Sum intensity of synapsin in posterior hilus increased at 7 DPI (**t**) compared to sham (**q**) then decreased at 30 DPI (**w**). Histograms of synapsin sum intensity (**z**, **bb**, **dd**) display the frequency of higher synapsin intensities at 7 DPI compared to sham and 30 DPI. **r**, **u**, and **x** display the merge of synapsin and MAP2 channels (scale bar = 25 μm). Data are represented as the percentage of total cells. Black vertical lines are drawn at the mean value for each experimental group. The shift in cell area or synapsin sum intensity is represented by colored horizontal bars, which indicate the percentage shift

To examine potentially subtler mossy cell pathology, we determined the area of the mossy cell somata as a means to measure cell hypertrophy (Fig. 3). In anterior hippocampal hilus (*F* = (2/275) 8.992, *p* = 0.0002), mossy cell soma size increased at 7 DPI (mean = 584.3 μm^2^, SEM ± 14.89) (*p* = 0.0004, *d* = 0.60) and 30 DPI (mean = 583.8 μm^2^, SEM ± 13.55) (*p* = 0.0009, *d* = 0.66) compared to sham (mean = 502.2 μm^2^, SEM ± 14.07). There was no statistical difference in mossy cell size between 7 DPI and 30 DPI (*p* = 0.997, *d* = 0.003). Histograms of mossy soma size in anterior hilus further illustrate mossy cell hypertrophy after mild TBI as the data distribution shifts rightward, indicating an overall soma area increase at 7 DPI and 30 DPI compared to sham (Fig. 3j, l, and n). Two-sample K-S tests also support a change in the mossy cell area data distribution; there is a significant difference in data distribution between sham and 7 DPI (*p* = 0.0003), and sham and 30 DPI (*p* = 0.0001), but not 7 DPI and 30 DPI (*p* = 0.4549).

In posterior hippocampal hilus (*F* = (2/996) 12.87, *p* < 0.0001), mossy cell soma size increased at 30 DPI (mean = 649.5 μm^2^, SEM ± 8.938) compared to sham (mean = 599.5 μm^2^, SEM ± 8.212) (*p* = <  0.0001, *d* = 0.32) and 7 DPI (mean = 594.0 μm^2^, SEM ± 7.853) (*p* = <  0.0001, *d* = 0.37). There was no statistical difference in mossy cell size between sham and 7 DPI (*p* = 0.8816, *d* = 0.04). Histograms of the mossy soma size in posterior tissue show a rightward shift of the data distribution at 30 DPI compared to sham and 7 DPI, further demonstrating mossy cell hypertrophy after mild TBI (Fig. 3y, aa, and cc). Two-sample K-S tests also support a change in the mossy cell area data distribution; there is no significant difference in distribution between sham and 7 DPI (*p* = 0.2359), but there is a significant difference between 7 DPI and 30 DPI (0.0001), and sham and 30 DPI (*p* = 0.0006).

### Synapsin-positive staining increased at 7 days after mild TBI

As there were significant changes in the mossy cell area, we postulated that local synaptic circuitry may have been remodeled after mild TBI [33]. Therefore, to histologically assess synaptic changes, we looked at the synaptic inputs to the mossy cells themselves. The sum intensity of synapsin, a presynaptic vesicle marker, was measured in a 30-μm radius circle ROI around each mossy cell. This sum intensity is a metric for the total amount of secondary antibody fluorescent molecules bound to synapsin I proteins and therefore a measure of synapsin expression. As such, synapsin staining was used as a surrogate to assess the synaptic connections between the mossy cells and the granule cell layer.

This analysis revealed that, in anterior hippocampal hilus (*F* = (2/217) 21.06, *p* < 0.0001), synapsin labeling increased over 1.5-fold at 7 DPI (mean = 3,886,994 AU, SEM ± 216,504) compared to sham (mean = 2,224,889 AU, SEM ± 149,115) (*p* = < 0.0001, *d* = 0.92). Synapsin labeling then decreased at 30 DPI (mean = 2,532,768 AU, SEM ± 182,770) compared to 7 DPI (*p* = <  0.0001, *d* = 0.76), effectively returning to baseline (sham levels). There was no statistical difference in synapsin labeling between sham and 30 DPI specimens (*p* = 0.6367, *d* = 0.25). Histograms of synapsin sum intensity reveal the frequency of greater synapsin labeling at 7 DPI compared to 30 DPI and sham. Specifically, we can see the synapsin sum intensity for sham specimens peaked around 2 × 10^6^ AU and began to steadily drop off to a maximum of 5 × 10^6^ AU. Thirty DPI specimens also reached a maximum for sum intensity at 5 × 10^6^ AU but seem to have a bimodal distribution of data with peaks at 2 × 10^6^ AU and 4 × 10^6^ AU. Notably, 7 DPI specimens had a less obvious data peak but had 33% of ROI synapsin sum intensities between 5 × 10^6^ AU and 8 × 10^6^ AU, while sham and 30 DPI had only 1% of their total data points in this higher range (Fig. 3k, m, and o). Two-sample K-S tests also supported this synapsin labeling change at 7DPI: sham and 7 DPI (*p* = 0.0001), as well as 7 DPI and 30 DPI (*p* = 0.0018), had significantly different data distributions, while sham and 30 DPI (*p* = 0.1556) did not.

In the posterior hippocampal hilus (*F* = (2/970) 55.23, *p* < 0.0001), synapsin labeling increased over 1.5-fold at 7 DPI (mean = 3,828,213 AU, SEM ± 225,361) compared to sham (mean = 2,095,759 AU, SEM ± 70,190) (*p* = <  0.0001, *d* = 0.53) yet decreased at 30 DPI (mean = 1,389,796 AU, SEM ± 117,217) compared to both 7 DPI (*p* = <  0.0001, *d* = 0.74) and sham (*p* = 0.0158, *d* = 0.46). Histograms of synapsin sum intensity again reveal the frequency of greater synapsin labeling at 7 DPI compared to 30 DPI and sham. Specifically, sham synapsin sum intensity reached a maximum at 6 × 10^6^ AU and 30 DPI sum intensity reached a maximum at 1 × 10^7^ AU, whereas 7 DPI had 17% of ROI synapsin sum intensities greater than 1 × 10^7^ AU and reached a maximum at 1.5 × 10^7^ AU (Fig. 3z, bb, dd). Two-sample K-S tests also supported this synapsin labeling change between experimental groups: sham and 7 DPI (*p* = 0.0001), sham and 30 DPI (*p* = 0.0001), and 7 DPI and 30 DPI (*p* = 0.0001) all have significantly different data distributions.

### Microglia density around mossy cells change following mild TBI

**Fig. 4:**
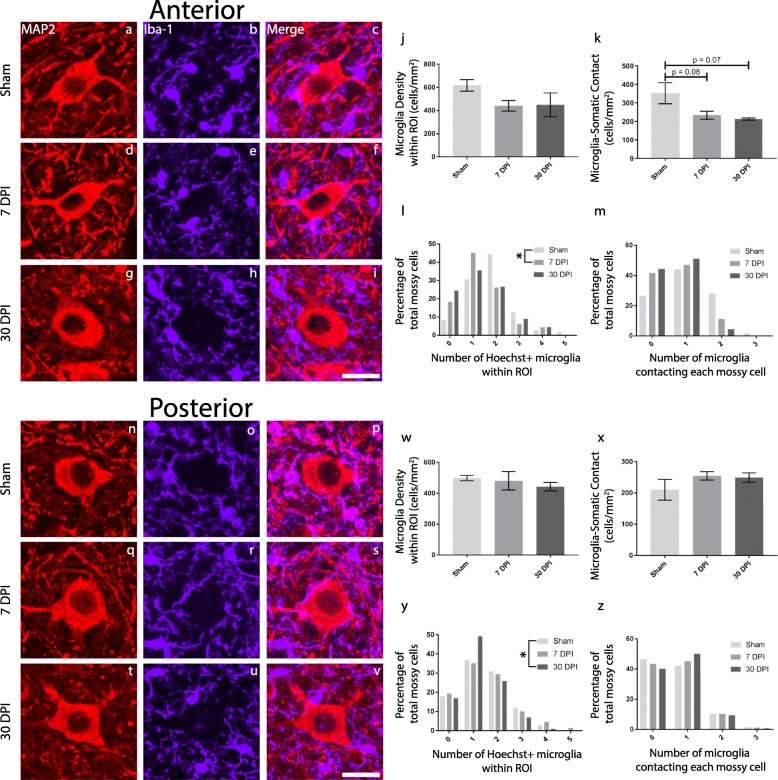
Microglia density proximal to mossy cells changes following mild TBI. To examine the migration of microglia after injury, we counted Iba-1-positive cells within the ROI around each mossy cell from sham, 7 DPI, and 30 DPI specimens in the anterior and posterior hippocampal hilus. Additionally, we also counted the Iba-1-positive cells that directly contacted the soma of each identified mossy cell. Representative images of anterior hilar mossy cells from sham (**a**), 7 DPI (**d**), and 30 DPI (**g**) specimens are shown, while **n**, **q**, and **t** correspond to the posterior hilar mossy cells of those animals (scale bar = 25 μm). Representative images of microglia activity in anterior hippocampal hilus from sham (**b**), 7 DPI (**e**), and 30 DPI (**h**) specimens are shown, while **o**, **r**, and **u** correspond to the posterior hilar microglia activity of those animals. Merged images of MAP2 and Iba-1 in anterior and posterior hilus are shown in **c**, **f**, and **i** and **p**, **s**, and **v** respectively. **j** There was a non-significant decrease in microglia density in anterior hilus at 7 DPI (*p* = 0.1377) and 30 DPI (*p* = 0.2258) compared to sham. Data represent mean +/− SEM. **l** A histogram of the number of Hoechst-positive microglia per mossy cell within this ROI in anterior hilus indicates a leftward shift of the data distribution for 7 DPI and 30 DPI compared to sham. **k** There was a nonsignificant decrease in the number of microglia on anterior hilar mossy cell somata at 7 DPI (*p* = 0.0889) and 30 DPI (*p* = 0.0794). **m** A histogram of the number of microglia contacting anterior hilar mossy cell somata demonstrates a leftward shift of the data distribution at 7 DPI and 30 DPI compared to sham. **w** There was no change in microglia density within the ROI at 7 DPI (*p* = 0.9573) and 30 DPI (*p* = 0.7254) compared to sham in posterior hilus. **x** There was no significant change in the number of microglia on posterior hilar mossy cell somata at 7 DPI (*p* = 0.3446) and 30 DPI (*p* = 0.5170). **y**, **z** Histograms of microglia density within the ROI and on mossy cell somata in posterior hilus did not reveal additional data trends

To examine the potential migration of microglia in the hilus after injury, we counted Iba-1-positive cells with visible Hoechst-positive nuclei within a 30-μm radius circle ROI around each mossy cell (Fig. 4). In anterior hippocampal hilus (*F* = (2/9) 2.648, *p* = 0.1247), there were no significant differences in ROI microglia density at 7 DPI (mean = 441.9 cells/mm^2^, SEM ± 45.45) (*p* = 0.1377, *d* = 1.74) and 30 DPI (mean = 449.3 cells/mm^2^, SEM ± 103) (*p* = 0.2258, *d* = 1.16) compared to sham (mean = 617.1 cells/mm^2^, SEM ± 49.94). Furthermore, there was no significant change between 7 DPI and 30 DPI (*p* = 0.9962, *d* = 0.05) (Fig. 4j). Using a histogram to provide further detail of ROI microglia density, we plotted the number of microglia around each mossy cell. We observed a leftward shift in the data distribution at 7 DPI and 30 DPI compared to sham suggesting that most mossy cells from 7 DPI or 30 DPI specimens had zero or one microglia within the ROI, while most mossy cells from sham specimens had two or more microglia within the ROI (Fig. 4l). Two-sample K-S tests reveal that the sample distribution of the number of ROI microglia was significantly different between sham and 7 DPI (*p* = 0.0095) but not between 7 DPI and 30 DPI (*p* = 0.9997) or sham and 30 DPI (*p* = 0.1693), which suggests that 7 DPI specimens have less microglia within the ROI more frequently compared to sham.

We then assessed the direct interaction of Hoechst-positive microglia with mossy cells (*F* = (2/9) 4.064, *p* = 0.0553). There were no significant differences in the number of microglia contacting mossy cells per square millimeter at 7 DPI (mean = 233.3 cells/mm^2^, SEM ± 21.81) (*p* = 0.0889, *d* = 1.36) and 30 DPI (mean = 212.8 cells/mm^2^, SEM ± 5.897) (*p* = 0.0794, *d* = 1.73) compared to sham (mean = 352.3 cells/mm^2^, SEM ± 56.66). There was no significant difference between 7 DPI and 30 DPI (*p* = 0.9223, *d* = 0.58) (Fig. 4k). We also plotted a histogram of the number of microglia directly contacting each mossy cell (Fig. 4m). Sham specimens had a normal distribution of data; approximately 40% of sham mossy cells have one contacting microglia while approximately 25% had zero and another 25% had two contacting microglia. In comparison, 7 DPI and 30 DPI specimens had a leftward shift of the data distribution; over 85% of 7 DPI or 30 DPI mossy cells have zero or one microglia contacting the soma. However, two-sample K-S tests revealed that the sample distribution of the number of microglia contacting each mossy cell was not significantly different between sham and 7 DPI (*p* = 0.1213), 7 DPI and 30 DPI (*p* = 0.9981), or sham and 30 DPI (*p* = 0.0684).

In the posterior hippocampal hilus (*F* = (2/9) 0.3085, *p* = 0.7420), there were no significant changes in ROI microglia density at 7 DPI (mean = 481 cells/mm^2^, SEM ± 59.72) (*p* = 0.9573, *d* = 0.18) and 30 DPI (mean = 442.9 cells/mm^2^, SEM ± 27.5) (*p* = 0.7254, *d* = 1.33) compared to sham (mean = 498.8 cells/mm^2^, SEM ± 17.67), nor was there a significant difference between 7 DPI and 30 DPI (*p* = 0.8471, *d* = 0.38) (Fig. 4w). A histogram of this data revealed that 30 DPI specimens tended to have one microglia within the ROI more frequently than 7 DPI and sham (Fig. 4y). Two sample K-S tests support this observation as there was a significant difference in the data distribution of microglia density between 7 DPI and 30 DPI (*p* = 0.0252), as well as sham and 30 DPI (*p* = 0.0237), but not between sham and 7 DPI (*p* = 0.9956).

In addition, there were no significant differences in microglia contacting mossy cells per square millimeter in the posterior hippocampal hilus (*F* = (2/9) 1.208, *p* = 0.3431) at 7 DPI (mean = 255.0 cells/mm^2^, SEM ± 13.54) (*p* = 0.3446, *d* = 0.87) and 30 DPI (mean = 249.4 cells/mm^2^, SEM ± 14.69) (*p* = 0.5170, *d* = 0.78) compared to sham (mean = 210.3 cells/mm^2^, SEM ± 33.19), nor was there a significant difference between 7 DPI and 30 DPI (*p* = 0.9842, *d* = 0.20) (Fig. 4x). A histogram of this data did not reveal additional trends (Fig. 4z). Finally, two-sample K-S tests suggest that the sample distribution is not different between sham and 7 DPI (*p* = 0.9960), or 7 DPI and 30 DPI (*p* = 0.9940), and sham and 30 DPI (*p* = 0.5162).

### Microglia density changes in additional hippocampal subfields over time post-injury

In order to put the changes related to hilar moss cells, synapses, and microglia in a broader context, we measured microglial morphological and density changes across various hippocampal subfields related to the dentate gyrus. To provide a wider temporal context, these analyses were performed in tissue sections from pig subjects at 3 DPI, 7 DPI, 30 DPI, and 1 YPI following mild TBI (compared to age-matched sham animals). First, semiquantitative assessment of microglial morphology using a graded scale in the ML, GCL, and hilus of the hippocampus (at both anterior and posterior levels) revealed an absence of overtly activated microglia. Indeed, blinded assessment did not detect any changes in microglia morphology across groups or over time.

**Fig. 5:**
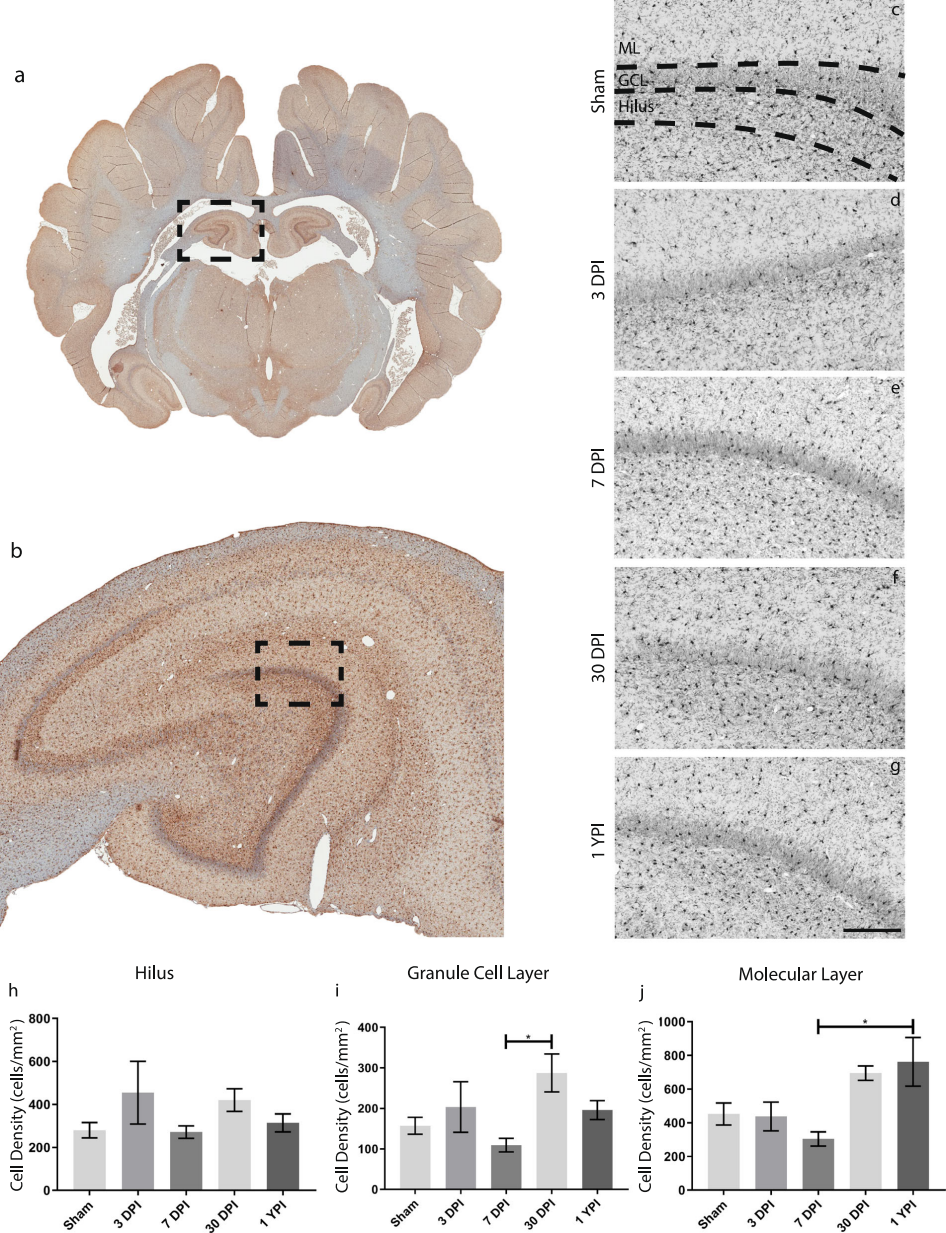
Microglia density increases in the anterior hippocampus over time following mild TBI. Microglia, stained with Iba-1, in pig coronal sections containing anterior hippocampus (**a**) with an enlarged call out box (**b**). The molecular layer (ML), granule cell layer (GCL), and Hilar region of the hippocampus were identified for each specimen and a representative image of Iba-1 staining for each experimental group is displayed (**c**–**g**) (scale = 200 μm). Microglia cell counts are quantified and graphed (**h**–**j**). Overall, biphasic trends can be observed as microglia tended to increase at 3 days post-injury (DPI), then decrease at 7 DPI, and finally increase again at chronic timepoints. **i** There is a significant increase in microglia from 7 DPI to 30 DPI in the GCL (*p* = 0.0312) and **j** a significant increase in microglia from 7 DPI to 1 year post-injury (YPI) in the ML (*p* = 0.0348)

Therefore, we next measured microglia density in the ML, GCL, and hilus (at both anterior and posterior levels) at 3 DPI, 7 DPI, 30 DPI, and 1 YPI. Microglial density was also measured in age-matched sham animals; however, there were no differences in any of the sham groups out to 1 YPI, so these animals were combined into a single sham group for statistical analysis. Across the ML, GCL, and hilus, the same general trends were observed: a modest, but not significant, increase in microglial density at 3 DPI; a return to baseline by 7 DPI; and a second, non-significant increase again at 30 DPI which, in most cases, was sustained (or re-elevated) out to 1 YPI. However, microglia density changes were only statistically significant in the GCL (*F* = (4/23) 2.880, *p* = 0.0454) and ML (*F* = (4/23) 3.402, *p* = 0.0252), but not the hilus (*F* = (4/23) 1.567, *p* = 0.2167) in anterior sections of hippocampus. Specifically, in anterior sections of hippocampal tissue, microglia density increased over 2.5-fold in both the GCL at 30 DPI (mean = 287.8 cells/mm^2^, SEM ± 46.85) compared to 7 DPI (mean = 109.5 cells/mm^2^, SEM ± 16.76) (*p* = 0.0312), as well as in the ML at 1 YPI (mean = 762.8 cells/mm^2^, SEM ± 144.6) compared to 7 DPI (mean = 304.5 cells/mm^2^, SEM ± 42.16) (*p* = 0.0348) (Fig. 5). All descriptive statistics and multiple comparison significance values between anterior hippocampus experimental groups are summarized in Tables 1 and 2, respectively.

**Table 1:** Microglia density in anterior hippocampal tissue. All values are reported as mean ± standard error mean, 95% confidence interval [lower 95% CI, upper 95% CI]

	Hilus	Granule cell layer	Molecular layer
Sham	280.2 cells/mm^2^ ± 35.53, 95% CI [202.7, 357.6]	157.2 cells/mm^2^ ± 20.80, 95% CI [111.9, 202.5]	452.6 cells/mm^2^ ± 65.50, 95% CI [309.8, 595.3]
3 DPI	455.1 cells/mm^2^ ± 146.1, 95% CI [− 9.7, 920.0]	203.4 cells/mm^2^ ± 62.30, 95% CI [5.155, 401.7]	437.7 cells/mm^2^ ± 85.33, 95% CI [166.1, 709.2]
7 DPI	271.6 cells/mm^2^ ± 28.94, 95% CI [191.2, 351.9]	109.5 cells/mm^2^ ± 16.76, 95% CI [62.94, 156.0]	304.5 cells/mm^2^ ± 42.16, 95% CI [187.4, 421.5]
30 DPI	420.2 cells/mm^2^ ± 52.69, 95% CI [193.5, 646.8]	287.8 cells/mm^2^ ± 46.85, 95% CI [86.18, 489.4]	695.1 cells/mm^2^ ± 42.96, 95% CI [510.2, 879.9]
1 YPI	314.4 cells/mm^2^ ± 42.06, 95% CI [133.4, 495.4]	195.8 cells/mm^2^ ± 23.38, 95% CI [95.23, 296.5]	762.8 cells/mm^2^ ± 144.6, 95% CI [140.8, 1385]

**Table 2:** Microglia density *p* values in the anterior hippocampus. All values displayed are the adjusted *p* values following a Tukey’s multiple comparisons test and Cohen’s *d* effect size

	Hilus	Granule cell layer	Molecular layer
Sham vs. 3 DPI	*p* = 0.2616, *d* = 0.77	*p* = 0.8291, *d* = 0.45	*p* > 0.9999, *d* = 0.07
Sham vs. 7 DPI	*p* > 0.9999, *d* = 0.08	*p* = 0.7631, *d* = 0.80	*p* = 0.6336, *d* = 0.82
Sham vs. 30 DPI	*p* = 0.5807, *d* = 1.25	*p* = 0.0939, *d* = 1.67	*p* = 0.3532, *d* = 1.38
Sham vs. 1 YPI	*p* = 0.996, *d* = 0.32	*p* = 0.9327, *d* = 0.64	*p* = 0.1481, *d* = 1.27
3 DPI vs. 7 DPI	*p* = 0.3651, *d* = 0.87	*p* = 0.3866, *d* = 1.02	*p* = 0.8579, *d* = 0.97
3 DPI vs. 30 DPI	*p* = 0.9978, *d* = 0.16	*p* = 0.6123, *d* = 0.80	*p* = 0.4665, *d* = 1.95
3 DPI vs. 1 YPI	*p* = 0.7211, *d* = 0.66	*p* > 0.9999, *d* = 0.08	*p* = 0.2462, *d* = 1.52
7 DPI vs. 30 DPI	*p* = 0.6435, *d* = 1.88	*p* = 0.0312, *d* = 2.82	*p* = 0.0916, *d* = 4.60
7 DPI vs. 1 YPI	*p* = 0.9943, *d* = 0.62	*p* = 0.5503, *d* = 2.21	*p* = 0.0348, *d* = 2.42
30 DPI vs. 1 YPI	*p* = 0.9009, *d* = 1.28	*p* = 0.5951, *d* = 1.43	*p* = 0.9935, *d* = 0.37

**Fig. 6:**
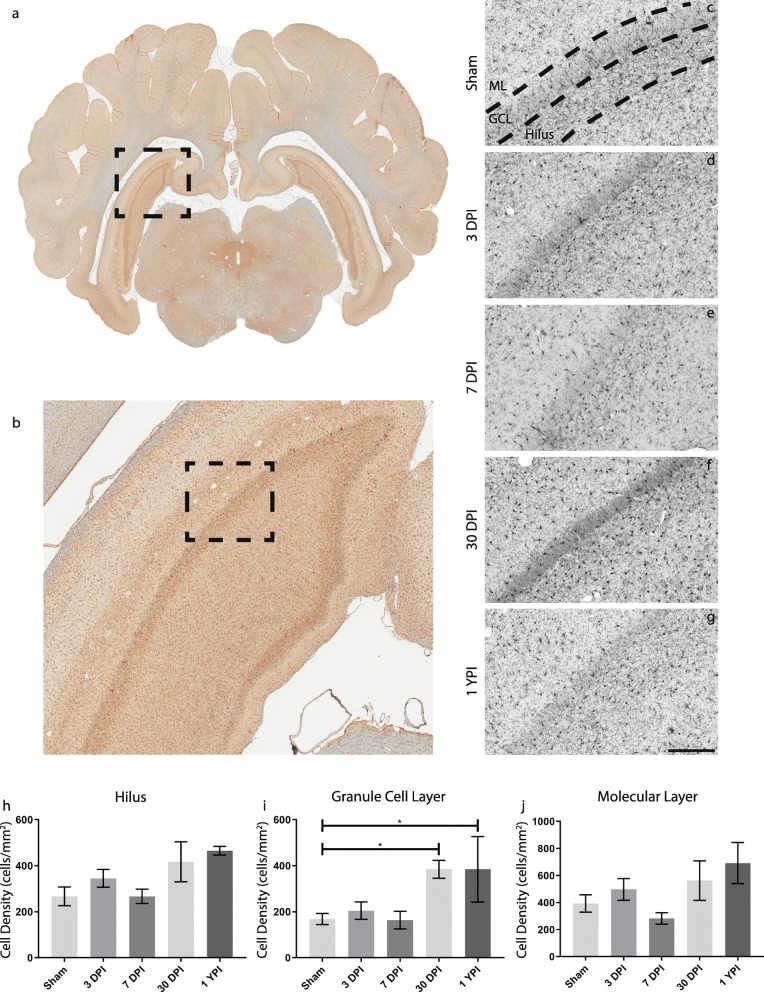
Microglia density increases in the posterior hippocampus over time following mild TBI. Microglia in pig coronal sections containing posterior hippocampus (**a**) with an enlarged call out box (**b**). The ML, GCL, and Hilar region of the hippocampus were identified for each specimen and a representative image of Iba-1 staining for each experimental group is displayed (**c**–**g**) (scale = 200 μm). Microglia cell counts are quantified and graphed (**h**–**j**). Like the anterior hippocampus, biphasic trends can be observed as microglia tended to increase at 3 DPI, then decrease at 7 DPI, and finally increase again at chronic timepoints. **i** There is a significant increase in microglia in the GCL at 30 DPI (*p* = 0.0365) and 1 YPI (*p* = 0.0364) compared to sham

In posterior sections of hippocampal tissue, microglia density changes were only statistically significant in the GCL (*F* = (4/22) 4.485, *p* = 0.0084), but not the ML (*F* = (4/23) 2.253, *p* = 0.0946) or the hilus (*F* = (4/22) 2.303, *p* = 0.0907). Microglia density increased over twofold in both the GCL at 30 DPI (mean = 384.6 cells/mm^2^, SEM ± 39.04) (*p* = 0.0365) and 1 YPI (mean = 384.7 cells/mm^2^, SEM ± 142.2) (*p* = 0.0364) compared to sham (mean = 168.5 cells/mm^2^, SEM ± 24.37) (Fig. 6). All descriptive statistics and multiple comparison significance values between posterior hippocampus experimental groups are summarized in Tables 3 and 4, respectively. Of note, the density of “microglia” was determined based on the expression of Iba-1^+^ cells; however, we cannot rule out that the increase is Iba-1-positive cells found at certain time points post-injury also includes a component of infiltrating peripheral macrophages, which could also be Iba-1 positive.

**Table 3:** Microglia density in the posterior hippocampal tissue. All values are reported as mean ± standard error mean, 95% confidence interval [lower 95% CI, upper 95% CI]

	Hilus	Granule cell layer	Molecular layer
Sham	267.1 cells/mm^2^ ± 40.93, 95% CI [177.9, 356.2]	168.5 cells/mm^2^ ± 24.37, 95% CI [115.4, 221.6]	393.6 cells/mm^2^ ± 64.13, 95% CI [253.9, 533.3]
3 DPI	345.1 cells/mm^2^ ± 38.46, 95% CI [222.7, 467.5]	205.1 cells/mm^2^ ± 37.88, 95% CI [84.57, 325.6]	497.6 cells/mm^2^ ± 80.47, 95% CI [241.5, 753.7]
7 DPI	267 cells/mm^2^ ± 31.04, 95% CI [168.2, 365.8]	164.0 cells/mm^2^ ± 38.35, 95% CI [41.95, 286.0]	282.9 cells/mm^2^ ± 42.38, 95% CI [165.3, 400.6]
30 DPI	417.2 cells/mm^2^ ± 86.95, 95% CI [43.07, 791.3]	384.6 cells/mm^2^ ± 39.04, 95% CI [216.6, 552.6]	562.8 cells/mm^2^ ± 146.3, 95% CI [− 66.69, 1192]
1 YPI	465.2 cells/mm^2^ ± 19.15, 95% CI [382.8, 547.6]	384.7 cells/mm^2^ ± 142.2, 95% CI [− 227.3, 996.7]	692.3 cells/mm^2^ ± 152.3, 95% CI [37.16, 1347]

**Table 4:** Microglia density *p* values in the posterior hippocampus. All values displayed are the adjusted *p* values following a Tukey’s multiple comparisons test and Cohen’s *d* effect size

	Hilus	Granule cell layer	Molecular layer
Sham vs. 3 DPI	*p* = 0.8046, *d* = 0.66	*p* = 0.9751, *d* = 0.45	*p* = 0.9076, *d* = 0.52
Sham vs. 7 DPI	*p* > 0.9999, *d* = 0.0008	*p* > 0.9999, *d* = 0.05	*p* = 0.8538, *d* = 0.63
Sham vs. 30 DPI	*p* = 0.3514, *d* = 1.00	*p* = 0.0365, *d* = 2.76	*p* = 0.7215, *d* = 0.70
Sham vs. 1 YPI	*p* = 0.1281, *d* = 1.85	*p* = 0.0364, *d* = 1.17	*p* = 0.2109, *d* = 1.20
3 DPI vs. 7 DPI	*p* = 0.8973, *d* = 1.12	*p* = 0.9824, *d* = 0.54	*p* = 0.562, *d* = 1.63
3 DPI vs. 30 DPI	*p* = 0.9389, *d* = 0.60	*p* = 0.2278, *d* = 2.50	*p* = 0.994, *d* = 0.31
3 DPI vs. 1 YPI	*p* = 0.7122, *d* = 2.02	*p* = 0.2275, *d* = 0.99	*p* = 0.7463, *d* = 0.89
7 DPI vs. 30 DPI	*p* = 0.5211, *d* = 1.30	*p* = 0.0921, *d* = 3.05	*p* = 0.3882, *d* = 1.46
7 DPI vs. 1 YPI	*p* = 0.258, *d* = 3.98	*p* = 0.0919, *d* = 1.21	*p* = 0.0922, *d* = 2.07
30 DPI vs. 1 YPI	*p* = 0.9889, *d* = 0.44	*p* > 0.9999, *d* = 0.001	*p* = 0.9415, *d* = 0.50

Additionally, all observed microglia density changes were homogenous within each defined hippocampal subregion; no distinct microglia clustering was observed.

## Discussion

A history of TBI is associated with cognitive impairment, such as short-term memory deficits and disrupted cognitive processing, with even a single so-called mild TBI potentially leading to long-term changes in memory performance and hippocampal structure [34]. After a single mild TBI using our pig model of closed-head rotational-acceleration, while there was no evidence of mossy cell loss, we found significant mossy cell hypertrophy at 7 DPI and 30 DPI in anterior (> 16% increase in mean cell area at each time; *p* = <  0.001 each) and 30 DPI in posterior (8.3% increase; *p* = <  0.0001) hippocampus. We also found dramatic increases in synapsin staining around mossy cells at 7 DPI in both anterior (74.7% increase in synapsin labeling; *p* = <  0.0001) and posterior (82.7% increase; *p* = <  0.0001) hippocampus. Interestingly, these alterations correlated with a significant change in microglia in proximity to mossy cells at 7 DPI in anterior and at 30 DPI in the posterior hippocampus via K-S tests. For broader context, while we found that there were significant increases in microglia density in the granule cell layer at 30 DPI (anterior and posterior) and 1 YPI (posterior only) and in the molecular layer at 1 YPI (anterior only), we found no significant changes in overall microglial density in the hilus at any of the time points evaluated post-injury. No overt changes in microglial morphology indicative of activation (i.e., shorter, thickened processes) were observed in any of the hippocampal subfields at any of the time points evaluated. Our hypothesis was that microglia would be more active in the hippocampal hilus and that there will be mossy cell loss following mild TBI. Based on our findings, we are compelled to reject this hypothesis.

In the current study, we initially examined the state of the mossy cells themselves. As noted, we hypothesized that mossy cells would be lost after mild TBI. Yet, unlike previous rodent studies, we did not observe any mossy cell loss in our mild TBI model—at the mild injury level that we applied [5–8]. Specifically, the number of mossy cells per unit area did not change between sham, 7 DPI, or 30 DPI. Nevertheless, subtler mossy cell changes were observed. Mossy cells hypertrophied at 7 DPI and 30 DPI in anterior tissue and at 30 DPI in posterior tissue. It is also unclear why the anterior and posterior mossy cell data are disparate. Mossy cell axonal projections are variable depending on their location within the hippocampus according to rodent literature, yet we are unsure if this translates to pig hippocampal architecture [30, 35]. It is possible that, similar to rodents, pig hilar mossy cell connections may be affected differently following mild TBI depending on their location within the hippocampus. Yet, some of the detected changes may be due to the differences in our injury models; the closed-head rotation acceleration injury model produces a diffuse brain injury while the rodent FPI model requires removal of part of the skull and generally results in tissue disruption [36]. Mossy cell loss may be seen in different injury models or at higher injury levels, but a single mild TBI in our pig model appears to induce a subtler mossy cell pathology. Future studies should extend these results across escalating injury levels to ascertain threshold loading parameters for mossy cell loss following closed-head rotational acceleration induced TBI.

We next examined the potential for synaptic changes to the mossy cells. In anterior and posterior tissue, we found an increase in synapsin I, a marker of pre-synaptic vesicles, staining at 7 DPI compared to both sham and 30 DPI. This increased synapsin is inconsistent with other hippocampal TBI literature. In a rodent controlled cortical impact model, synapsin I expression decreased in the ipsilateral hippocampus at 7 and 21 DPI [37, 38]. Synapsin I loss has been documented even at 24 h after TBI, with researchers suggesting that synapsin I loss is a result of increased oxidative stress [39]. However, research on the mossy cells themselves has found enhanced action potential discharges in response to perforant path stimulation after rodent FPI, as researchers have hypothesized that mossy cells may be vulnerable to injury as a result of presynaptic mechanisms [5, 40]. In a mouse model of temporal lobe epilepsy, Zhang et al. observed a reduction of input resistance to mossy cells, as well as a frequency increase of miniature postsynaptic currents in epileptic mice, indicating an overall increase in excitatory synaptic input to mossy cells [33]. Interestingly, Jinde et al. used a transgenic mouse line to ablate mossy cells, which resulted in granule cell excitability but no detection of epileptiform activity [41]. In contrast, Bui et al. optogenetically inhibited mossy cells in a mouse model of temporal lobe epilepsy and observed that spontaneous electrographic seizures progressed into convulsive seizures [42]. Yet, this does not fully explain our detected decrease in synapsin at 30 DPI compared to 7 DPI. Future experiments with our current model should conduct whole-cell electrophysiology recordings to characterize potential functional changes to mossy cells in conjunction with the observed histological increase in presynaptic inputs. Nonetheless, our findings show that elements of circuit remodeling occur absent mossy cell loss following a single mild TBI. This circuit level disruption is supported by previous research from our group. In ex vivo hippocampal slices, dentate gyrus and CA1 recordings displayed reduced axonal function, regional hyperexcitability, and elevated excitatory post-synaptic potential at 7 DPI versus sham animals. These physiological changes occurred absent neuronal or axonal degeneration [4].

We also sought to examine microglia density proximal to hilar mossy cells, which are thought to be prone to damage and potential phagocytosis following TBI [5–8]. In addition, microglia may prune mossy cell synapses and therefore play a role in the decrease in presynaptic input at 30 DPI versus the stark increase in presynaptic input at 7 DPI. For instance, Eyo et al. noted that glutamate induced microglia to extend their processes towards hippocampal neurons and that knocking out the P2Y12 receptor in microglia worsened seizure behavior in a rodent model of epilepsy [14]. As such, we counted microglia density within a 30-μm radius circle around each mossy cell. While we did observe a trend of lower microglia densities at 7 DPI and 30 DPI compared to sham in anterior tissue, it was not statistically significant. We then assessed the direct interaction of microglia with mossy cells and found no significant differences in the number of microglia contacting mossy cells at 7 DPI (*p* = 0.0889) and 30 DPI (*p* = 0.0794) compared to sham. There was no noticeable trend for microglia activity changes in posterior tissue. However, K-S tests detected a significant change in the sample distribution of microglia within the ROI at certain time points. Therefore, future studies should closely monitor the activity of microglia, potentially by quantifying the alteration of synaptic components between 7 DPI and 30 DPI. Previous research using this pig model of injury noted an increase in microglia activity proximal to acutely injure neurons exhibiting plasma membrane damage [22]. However, the dye injection used to track membrane permeability is a terminal procedure, and thus, incompatible with a chronic study. Moreover, this previous work did not select specific anatomically defined cells or regions as was done in the current study, but rather used a sensitive marker of subtle neuronal injury to direct examinations of microglial morphology and density. Therefore, investigations focusing on regions showing marked pathology may be necessary to see large effect sizes in closed-head diffuse TBI that lacks any macro-scale injury epicenter.

To explore a potential role of microglial activation in mossy cell hypertrophy and synaptic remodeling, we next expanded our examination of microglial responsiveness to larger areas in and around the hilar region of the hippocampus. We initially hypothesized that microglia activity would increase in the hilus following mild TBI. However, our cell density counts indicate a more complex microglial response throughout the dentate gyrus. In anterior sections, there was a significant increase in cell density at 30 DPI compared to 7 DPI in the granule cell layer and a significant increase at 1 YPI compared to 7 DPI in the molecular layer. In posterior sections, there was an increase at 30 DPI and 1 YPI compared to sham in the granule cell layer. These examples of continuing microglia density changes may correspond to chronic phases of inflammation post-injury that previous literature has suggested; however, as noted, there were not morphological changes in microglia which runs contrary to conventionally accepted definitions of “neuroinflammation” that generally features an increased incidence of reactive, ameboid microglia. Our observed changes in microglia density without changes in microglia morphology are dissimilar to the noted neuroinflammatory response in rodents subjected to fluid percussion injury and other models of pig TBI. Of note, Gorse and Lafrenaye examined rat and pig thalamus following central fluid percussion injury. At 1 DPI, rats displayed a decrease in microglial process interactions around amyloid precursor protein stained, injured axons while pigs displayed an increase in microglial interactions with injured axons compared to sham [43]. Components of neuroinflammatory response include but are not limited to driving neuronal cell death, synaptic pruning, release of growth signals, and sequestering pathological debris [18, 44–47]. More investigation is needed to determine what phenotype and behavior microglia are prioritizing in this model of mild TBI. A review on neuroinflammation after TBI by Simon et al. summarizes that pathological proliferation of microglia is generally observed at 72 h after injury and peaks at 3 months, which is not inconsistent with our findings [48]. Additionally, chronically activated microglia were observed from 2 weeks to many years after TBI in human post mortem tissue, though this microglial response may also be influenced by several factors including age, sex, mechanism and degree of injury, and secondary insults [48]. Furthermore, rodent studies have noted an increase in activated microglia density at subacute and chronic time points starting at 1 week and up to 52 weeks after injury [23], which is also consistent with our findings although the method of inducing injury, the severity of the injury, and the experimental species are different than the current study. Future studies could investigate if these cells are pathological or detrimental to neuronal health or if these cells are playing a role in environmental support and synaptic remodeling/health.

Altered structure and function of mossy cells post-injury is relevant to one hypothesis suggesting that surviving mossy cells contribute to hyperexcitability and potential memory deficits following TBI [5]. This so-called irritable mossy cell hypothesis proposes that surviving mossy cells can grow aberrant axonal processes after TBI that projects into the granule cell layer creating excessive synaptic connections, as well as a positive excitable feedback loop to the hilus, which may contribute to the development of post-traumatic epilepsy [5, 9]. Moreover, mossy cell hypertrophy has been recorded in rodent models of epilepsy with some researchers theorizing that injured mossy cells may develop more cell machinery, and thus a larger cell body, to support axon sprouting [33, 49]. However, more research is needed to determine if these rodent model results translate to our pig model of mild TBI or human TBI. Future pig TBI studies should characterize this potential aberrant sprouting through immunohistochemistry or through viral axon tracers following injury.

Moreover, exploring the mechanisms that prime microglia responses, such as complement system activation and cytokine production, may be a crucial step in identifying therapeutic targets. Future measurements of changes in gene expression, which may quantify a wide array of inflammatory and synaptic RNA markers, followed by in situ hybridization, may provide an appropriate spatial resolution within whole sections of hippocampal tissue. These experiments would help confirm the potential inflammatory and synaptic alterations observed in the current study. Additional studies should also explore the significant increase in microglial density in the GCL at chronic time points. The increase in GCL microglia may be a response to the 7 DPI mossy cell synapsin levels. Microglia may be regulating GCL neurons themselves as granule cells provide much of the presynaptic inputs to the mossy cells. In particular, we can count the number of granule cell neurons, survey for ectopic neurons, and histologically assess perforant path inputs to the GCL and aberrant sprouting from the GCL after mild TBI. Moreover, while our current study only utilized postmortem structural markers, selective positron emission tomography (PET)- or magnetic resonance imaging (MRI)-based imaging methods would allow us to track putative neuroinflammation in a single subject over time, providing additional insight on the activity of microglia between our terminal time points. Finally, future studies should examine the effects of repetitive injuries on hippocampal alterations as microglia become more reactive and ameboid after repetitive injuries relative to single injuries [22]. Exposure to repetitive injuries may increase a patient’s risk for hippocampal dysfunction.

## Conclusions

Our pig model of closed-head rotational acceleration-induced diffuse TBI replicates key features of clinical TBI, including the biomechanical loading parameters underlying the pathogenesis of mild TBI and subsequent neuropathology in humans. Unlike rodents, pigs have a large brain mass, gyrencephalic brain architecture, and a gray to white matter ratio similar to humans [50–52]. This is particularly important because diffuse axonal injury in white matter is believed to be the principal pathology of closed-head diffuse TBI [53, 54]. The injury levels employed in this study were designed to biomechanically mimic levels associated with concussion in humans, and it is interesting that we did not observe mossy cell loss as shown in rodent preclinical models, but rather we found hilar mossy cell hypertrophy at 7 DPI and 30 DPI and an increase in synapsin staining at 7 DPI. This may be indicative of subtle mossy cell damage in concussion, aberrant sprouting between the mossy cells and granule cells, and potential hippocampal network hyperexcitability. However, the decrease in synapsin staining at 30 DPI may also indicate compensatory pruning, which would facilitate the removal of excess synaptic connections after TBI-induced damage. We also observed microglia density changes in the granule cell layer and molecular layer, but not the hilus, at certain time points following mild TBI in a clinically translatable model. Future studies should examine microglial modification of synaptic components and electrophysiological input and output changes related to mossy cells to confirm physiological circuitry changes. These studies would provide insight into the nexus of hippocampal circuit remodeling and neuroinflammatory processes, and thus may lend insight into hippocampal-related functional deficits following mild TBI.

## Data Availability

The datasets used during the current study are available from the corresponding author on reasonable request.

## Abbreviations

DAB: 3,3′-Diaminobenzidine
DPI: Days post-injury
GCL: Granule cell layer
Iba-1: Ionized calcium-binding adapter molecule
ML: Molecular layer
MRI: Magnetic resonance imaging
NBF: Neutral buffered formalin
PET: Positron emission tomography
TBI: Traumatic brain injury
YPI: Year post-injury
